# Study on Mechanical Properties and Curing Reaction Mechanism of Alkali-Activated-Slag Solidified Port Soft Soil with Different Activators

**DOI:** 10.3390/ma18071583

**Published:** 2025-03-31

**Authors:** Wenjun Hu, Han Zhang, Yu Cheng, Yi Xue, Yutong Han, Jianghua Jia, Kun Li, Zhifeng Zhang

**Affiliations:** 1 School of Transportation Engineering, Shandong Jianzhu University, Jinan 250101, China; zhang_h007@163.com (H.Z.); chengyu200111@163.com (Y.C.); 2023155134@stu.sdjzu.edu.cn (Y.X.); jrcl498498498@163.com (Y.H.); huayv20010927@163.com (J.J.); 15610138217@163.com (K.L.); 2 Key Laboratory of Building Structural Retrofitting and Underground Space Engineering (Shandong Jianzhu University), Ministry of Education, Jinan 250101, China; 3 Ural International Rail Transit College, Shandong Polytechnic, Jinan 250104, China

**Keywords:** AAS-solidified soil, orthogonal test, macroscopic mechanical properties, SEM-EDS, TG-DTG, FT-IR, microscopic reaction mechanism

## Abstract

The application of alkali-activated slag (AAS) cementing material to the curing of soft soil foundations has a good engineering application prospect and is economical and environmentally friendly. In this study, three different activators (Na_2_O·nSiO_2_, NaOH, Ca(OH)_2_) were used to alkali-activate slag powder to solidify and improve soft soil in inland port areas. In order to explore the mechanical properties and strength formation mechanism of AAS-solidified soil under different activators, mechanical properties, and microscopic tests were carried out. Firstly, with unconfined compressive strength as the evaluation index, an orthogonal test of three factors, such as the type of activator, the amount of activator, and the amount of slag powder, was designed. Then, the unconfined compressive strength, resilience modulus, shear strength, and compression modulus of AAS-solidified soil were tested with the three activators under optimal dosage. Finally, phase composition, SEM-EDS, TG-DTG, and FT-IR analyses were carried out with the three AAS-solidified soils. The results show the following: (1) The factors affecting the unconfined compressive strength of AAS-solidified soil are ordered as follows: the type of activator > the amount of activator > the amount of slag powder. In addition, the optimal factors were as follows: activator type: Na_2_O·nSiO_2_; amount of activator: 3%; and amount of slag powder: 20%. (2) In considering the macroscopic mechanical properties, the effect of the activator is Na_2_O·nSiO_2_ > NaOH > Ca(OH)_2_, and the Na_2_O·nSiO_2_ AAS-solidified soil has good early strength. (3) The hydration products of AAS are mainly C-A-S-H gel, N-A-S-H gel, and C-S-H gel, which increase the strength and cohesion of solidified soil. The results show that AAS-solidified soil with 0.7-modulus Na_2_O·nSiO_2_ as the activator has good engineering characteristics and can be used for curing soft soil foundations.

## 1. Introduction

The soft soil foundation in an inland port area has poor bearing capacity, high compressibility, and low shear strength. The soft soil foundation is prone to settlement deformation and other problems without treatment [1,2]. Alkali-activated slag (AAS) cementing material is an environmentally friendly cementing material produced with slag powder using an alkaline activator [3]. In recent years, more and more scholars have begun to pay attention to the development and properties of alkali-activated slag/steel slag cementing materials [4]. Some scholars have also studied the effects of adding modifiers such as latex and nano-silica [5,6,7] or high-temperature conditions [8] on the properties of AAS cementing materials, and some scholars have used AAS, fly ash, and coal gangue as solidified materials [9,10] for solidified soil [11,12,13]. Liu Lin et al. [14] prepared AASC fluid-solidified soil using alkali-activated slag cement instead of Portland cement to solidify sludge, and they studied the strength formation mechanism of solidified soil using SEM and EDS tests. Zhu Yue [15] used a geopolymer made of alkali-activated steel slag and cement instead of cement to produce mixing piles to reinforce the soft soil foundation. Yi Yaolin et al. [16] used three activators, NaOH, Na_2_SO_4_, and Na_2_CO_3_, to alkali-activate mineral powder + calcium carbide slag and compared its unconfined compressive strength. Liu Gang et al. [17] studied the compressive and flexural strength of an alkali-activated slag powder–fly ash geopolymer and analyzed the hydration process via XRD, SEM, EDS, and DTG. Yang Xiaoyun et al. [18] found through orthogonal tests that the influencing factors of geopolymer strength are, in order, slag content, alkali content, and the Na_2_O·nSiO_2_ modulus, and they clarified the strength development mechanism of a geopolymer through XRD, FTIR, and SEM-EDS tests.

After several rounds of tentative tests, it was found that some activators had a poor excitation effect on the selected slag powder, such as neutral salt Na_2_SO_4_, alkaline salt Na_2_CO_3_, NaHCO_3_, high-modulus Na_2_O·nSiO_2_, and weak acid CH_3_COOH. The OH^−^ in NaOH and Ca(OH)_2_ can effectively stimulate the activity of slag. The Na_2_O·nSiO_2_ with a suitable modulus adjusted by NaOH can not only provide OH^−^ but also introduce a silicon source so that more cementitious materials are produced after the activation of slag. Moreover, compared with other activators, the three activators have no irritating odor during the mixing process. Therefore, Na_2_O·nSiO_2_ with a suitable modulus, NaOH, and Ca(OH)_2_ were selected for the alkali excitation of slag powder to solidified soft soil in a port area. When preparing the sample, the slag powder and the soil were first fully mixed, and then the activator was dissolved in water to prepare a solution, which was then stirred for more than 3 min until the solution was transparent. The solution was added to the slag powder and the soil and mixed for more than 5 min to prepare the AAS-solidified soil. The optimal ratio of AAS-solidified soil was obtained by conducting an orthogonal test, and its mechanical properties and microscopic reaction mechanism under different activators were analyzed comprehensively.

## 2. Materials and Methods

### 2.1. Raw Material

**Figure 1:**
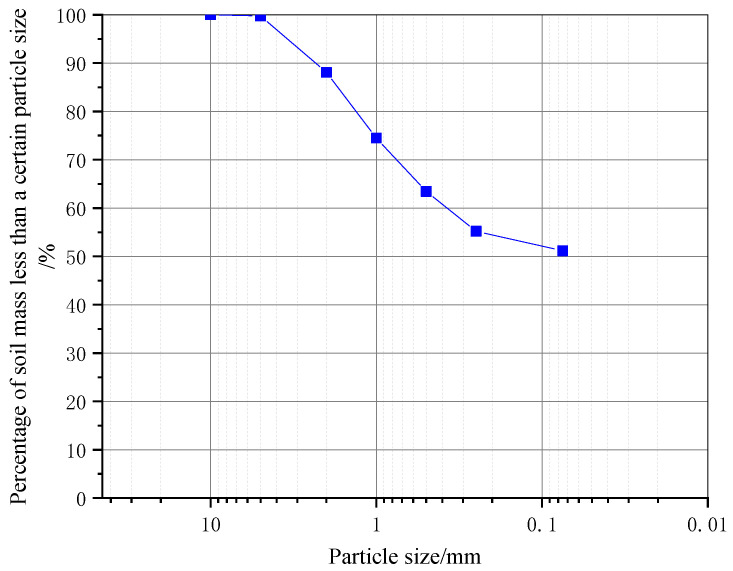
Grain size distribution.

The appearance and basic properties of the raw materials are shown in Table 1, Table 2, Table 3 and Table 4. The soil samples used in this study were taken from the main port area of Jinan Port. The particle distribution curve is shown in Figure 1. The content of the medium-fine-grain group (<0.075 mm) is 51.15%. In the coarse-grain group (2~60 mm), the content of sand (0.075~2 mm) is 36.92%, and the content of gravel (2~60 mm) is 11.93%. Since the mass of fine-grained soil particles is greater than 50% of the total mass, the soil samples taken according to the Standard for Soil Test Methods (JTG 3430-2020) [19] were fine-grained soil. The plasticity index I_P_ is 12.14, where 10 < I_P_ ≤ 17; the liquid limit is 30.61% < 50%; and the soil sample was named low-liquid-limit silty clay.

The metal oxide content of slag powder was analyzed using an X-ray fluorescence spectrometer (XRF, Rigaku ZSX Primus III+).

The Na_2_O·nSiO_2_ used in the test was Yourui-brand liquid Na_2_O·nSiO_2_, a colorless transparent liquid, slightly viscous; the NaOH and Ca(OH)_2_ used in the experiment were all analytically pure. The appearance and essential property indicators of the raw materials are shown in Table 1, Table 2, Table 3 and Table 4.

### 2.2. AAS Matching Design

#### 2.2.1. Mixing of Sodium Silicate Modulus

The modulus (nSiO_2_/nNa_2_O) is an important parameter of Na_2_O·nSiO_2_. The modulus of Na_2_O·nSiO_2_ was prepared with analytically pure NaOH using the following formulas and equations.

(1) $$n=\frac{m_{0}}{m}$$

In the formula, n is the amount of matter; m_0_ is the mass of the substance (g); and *m* is the molar mass of the matter (60 g/mol for SiO_2_ and 62 g/mol for Na_2_O).

(2) $$M=\frac{n(SiO_{2})}{n(Na_{2}O)}$$

In the formula, M is the Na_2_O·nSiO_2_ modulus, and n is the amount of matter.

The conversion equation between NaOH and Na_2_O is as follows:

(3) $$Na2O\cdot SiO_{2}+2NaOH⇌Na2O\cdot SiO_{2}+Na_{2}O+H_{2}O$$

Per 100 g of Yourui liquid Na_2_O·nSiO_2_, the equation is as follows:

(4) $$m_{n}=10.96\frac{M_{0}}{M_{n}}-10.96$$

In the formula, m_n_ is the mass (g) of NaOH that should be added when the modulus of Na_2_O·nSiO_2_ is n; M_0_ is the modulus of Youri liquid Na_2_O·nSiO_2_ = 3.3; and M_n_ is the required modulus n.

Specifically, when Na_2_O·nSiO_2_ with a modulus of n is needed, every 100 g of Youri liquid Na_2_O·nSiO_2_ with a modulus of 3.3 needs to be analyzed and purified with m_n_ g of NaOH so that Na_2_O·nSiO_2_ with a modulus of n of (100 + m_n_) g can be obtained.

#### 2.2.2. Optimum Modulus Test Scheme for Na_2_O·nSiO_2_

To explore the influence of the Na_2_O·nSiO_2_ modulus on AAS-solidified soil, the Na_2_O·nSiO_2_ modulus was optimized using an experimental design, as shown in Table 5. The original soil sample was mixed with slag powder and excitation material in an extrinsic manner, and cylinder specimens of h5 cm × Φ5 cm were prepared. Unconfined compressive strength tests were then carried out after curing for 7 days.

#### 2.2.3. Orthogonal Test Design of AAS-Solidified Soil

The Na_2_O·nSiO_2_ with best modulus value, NaOH, and Ca(OH)_2_ were separately administered different dosages of slag powder to investigate the strength properties of different AAS-solidified soils. An orthogonal test design was carried out. A, B, and C were used to represent three influencing factors: the type of activator, the amount of activator, and the amount of slag powder. Three different levels were designed for each influencing factor, and the values of each level showed equal gradient changes. The specimens were prepared according to the ratios in Table 6, and the unconfined compressive strength at the curing ages of 7 d and 28 d was measured.

### 2.3. Test Method

The unconfined compressive strength test was carried out according to the Standard for Soil Test Methods (JTG 3430-2020) [19]. The resilient modulus test, rapid consolidation test, and rapid shear test were carried out on the AAS-solidified soil under the optimal ratio, and the soil’s mechanical properties under the three activators were obtained.

To analyze the effect of different stimulants and curing ages on the hydration product, the solidified soil was dried to constant weight, and X-ray diffraction (XRD) test was carried out with a scanning range of 5~80° and a scanning speed of 2°/min. Then, Jade 6.0 software was used for analysis. The SEM-EDS test was carried out on the AAS-solidified soil with a scanning electron microscope (SEM) and energy dispersive spectrometer (EDS), and the morphology and element content of the product was observed and measured. The infrared spectrum of AAS-solidified soil was measured using a Fourier transform infrared spectrometer (FTIR, type: Thermo Fisher Scientific Nicolet S20, Waltham, MA, USA). The wave number range was 400~4000 cm^−1^, and the molecular structure and chemical bond of the hydration products were analyzed. A Thermal Gravimetric Analyzer (TG, model: Rigaku TG/DTA 8122) was used for thermogravimetric analysis. The test temperature was 30~800 °C, and the heating rate was 10 °C/min. The relationship between the mass loss of solidified soil and temperature was measured.

## 3. Results and Discussion

### 3.1. Optimum Modulus of Na_2_O·nSiO_2_

**Figure 2:**
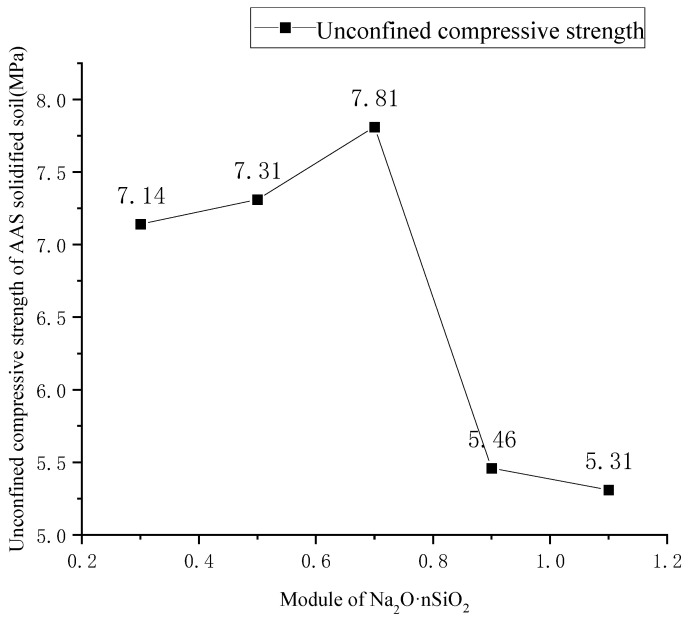
Influence of Na_2_O·nSiO_2_ modulus on unconfined compressive strength of AAS-solidified soil.

The unconfined compressive strength of the AAS-solidified soil was tested according to the values in Table 5, and the unconfined compressive strength of AAS-solidified port soft soil under different Na_2_O·nSiO_2_ modulus was obtained. As shown in Figure 2, when the modulus of Na_2_O·nSiO_2_ is 0.7, the unconfined compressive strength of the solidified soil is the highest, at 7.81 MPa, meaning that the best modulus of Na_2_O·nSiO_2_ is 0.7.

### 3.2. Analysis of Orthogonal Test Results

The orthogonal test results of the unconfined compression at 7 d and 28 d are shown in Table 7.

A range analysis was carried out on the test results in Table 7, and the relationship between the unconfined compressive strength of the AAS-solidified soil, and the three factors (activator type, activator content, and slag powder content) was explored, as shown in Table 8 and Figure 2.

**Figure 3:**
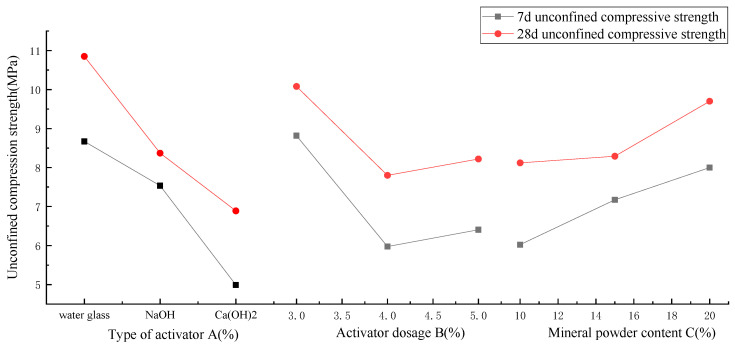
Diagram of AAS-solidified soil’s unconfined compressive strength and three factors.

As seen from Table 8 and Figure 3, the order of the factors affecting the unconfined compressive strength of AAS-solidified soil is activator type A > activator content B > slag powder content C. The changing trend of unconfined compressive strength at the curing ages of 7 d and 28 d is the same, but the unconfined compressive strength at 28 d is significantly higher than that at 7 d. The optimal factors are Na_2_O·nSiO_2_ as the activator, 3% as the activator content, and 20% as the slag powder. The type of activator has the most significant influence on AAS-solidified soil and is the main factor in improving strength. The unconfined compressive strength of the cured soil at 7 d is 1.15 and 1.73 times that of NaOH and Ca(OH)_2_, respectively, when the activator is Na_2_O·nSiO_2_, and at 28 d, it is 1.29 and 1.57 times that of NaOH and Ca(OH)_2_, respectively. That is, Na_2_O·nSiO_2_ has the best excitation effect, followed by NaOH and Ca(OH)_2_. When Na_2_O·nSiO_2_, NaOH, or Ca(OH)_2_ is used as the activator, the unconfined compressive strength of solidified soil at 28 d is 25%, 11%, or 38% higher, respectively, than that at 7 d. Based on the unconfined compressive strength of cured soil at 7 d and 28 d, Na_2_O·nSiO_2_ > NaOH > Ca(OH)_2_. The content of the activator is also an essential factor affecting the unconfined compressive strength of solidified soil. It can be seen from Figure 3 that the unconfined compressive strength of solidified soil is the best when the content of the activator is 3%, and the strength of solidified soil at 7 d is 48% and 38% higher than that when the content of the activator is 4% and 5%, respectively. It can be seen that a greater amount of activator does not mean that the unconfined compressive strength of solidified soil is higher. Finally, although slag powder is the primary curing agent, the main curing effect is the gelling material generated after the alkali excitation of slag powder. Hence, the content of slag powder is not the main factor for improving the strength of solidified soil. The unconfined compressive strength of the solidified soil with 20% slag powder increased by 38% compared with 10% with slag powder at 7 d, and the unconfined compressive strength at 28 d increased by 19%. Moreover, if the slag powder content is too high, the hydration speed will be too fast and more difficult to mix. At the same time, considering economic factors, the optimal content of 15% slag powder was selected.

### 3.3. Mechanical Properties of AAS-Solidified Soils

According to the orthogonal test results, the optimal settings involve stimulating 15% slag powder with 3% Na_2_O·nSiO_2_ (modulus of 0.7) with unconfined compressive strength as the evaluation index. In order to evaluate the mechanical properties of AAS-solidified soil under different activators, the unconfined compressive strength, resilience modulus, shear strength, and resilience modulus of the three kinds of activators were tested.

#### 3.3.1. Unconfined Compressive Strength

**Figure 4:**
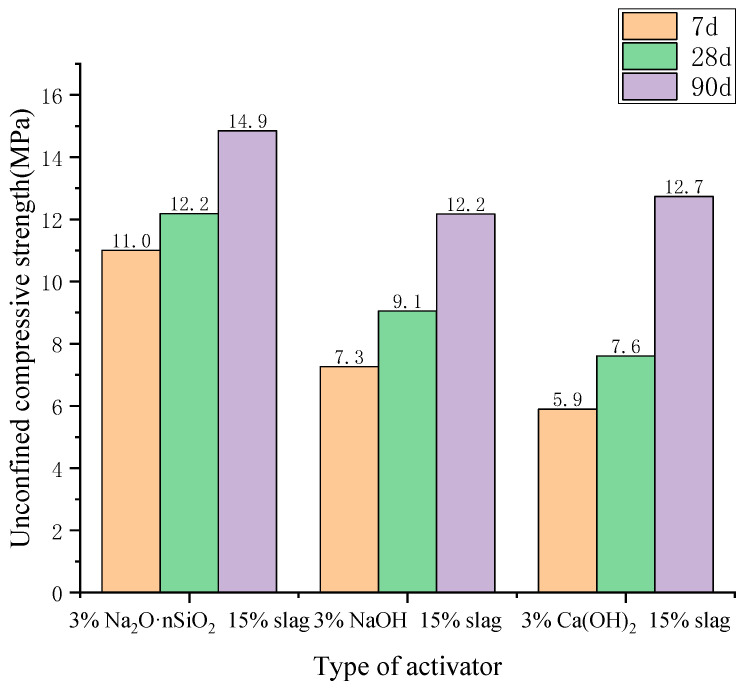
Unconfined compressive strength of AAS-solidified soil with different activators.

The influence of different activator types on the unconfined compressive strength of AAS-solidified soil is shown in Figure 4. When Na_2_O·nSiO_2_, NaOH, or Ca(OH)_2_ was used as the activator, the unconfined compressive strength of the cured soil at 7 d was 11.00 MPa, 7.3 MPa, and 5.9 MPa, respectively. At 28 d, the compressive strength increased slightly and was 12.2 MPa, 9.1 MPa, and 7.6 MPa, respectively, meaning that it increased by 10.7%, 24.63%, and 28.94%, respectively, compared with that at 7 d. Moreover, the compressive strength at 90 d continued to increase, with strengths of 14.85 MPa, 12.17 MPa, and 12.73 MPa, respectively, indicating that the unconfined compressive strength had increased by 35.45%, 67.12%, and 115.25%, respectively, compared with that at 7 d. It can be seen that the type of activator has a significant effect on the unconfined compressive strength, and the exciting impact of Na_2_O·nSiO_2_ is the best. In addition, the low compressive strength growth rate of Na_2_O·nSiO_2_ under different curing ages indicates that the alkali excitation of Na_2_O·nSiO_2_ results in early strength formation.

#### 3.3.2. Rebound Modulus

**Figure 5:**
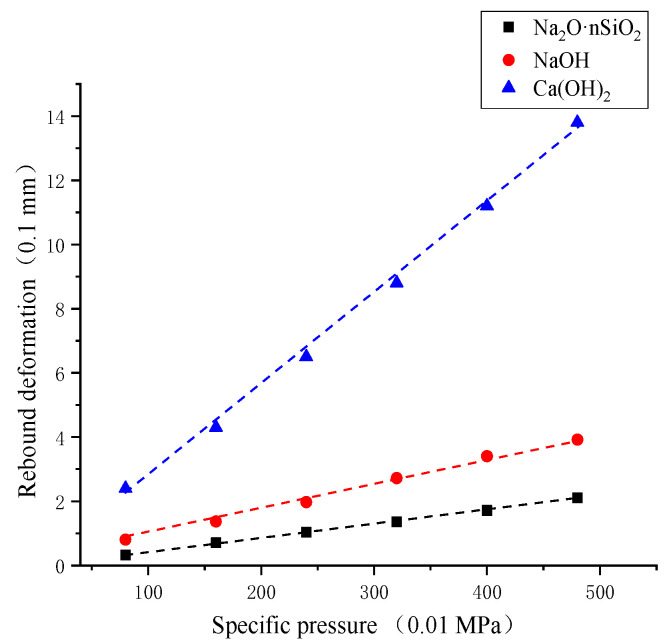
Relation curve of unit pressure–rebound deformation.

**Figure 6:**
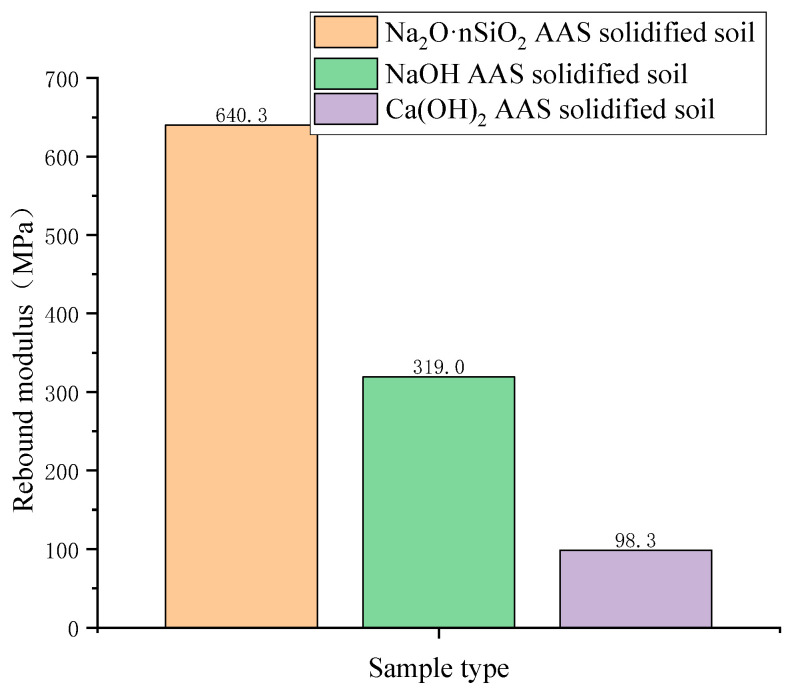
Resilience modulus of AAS-solidified soil under different activators.

The relationship between the unit pressure and rebound deformation of the AAS-solidified soil under different activators is shown in Figure 5. Under the same unit pressure, the deformation amount is the largest when the activator is NaOH and the smallest when the activator is Na_2_O·nSiO_2_. The resilience modulus of AAS-solidified soil is inversely proportional to the deformation. In comparing the results of the resilience modulus (Figure 6), that of Na_2_O·nSiO_2_-solidified soil is the largest, and that of Na_2_O·nSiO_2_-solidified soil is 2 times that of NaOH AAS-solidified soil and 6.5 times that of Ca(OH)_2_ AAS-solidified soil.

#### 3.3.3. Shear Strength

**Figure 7:**
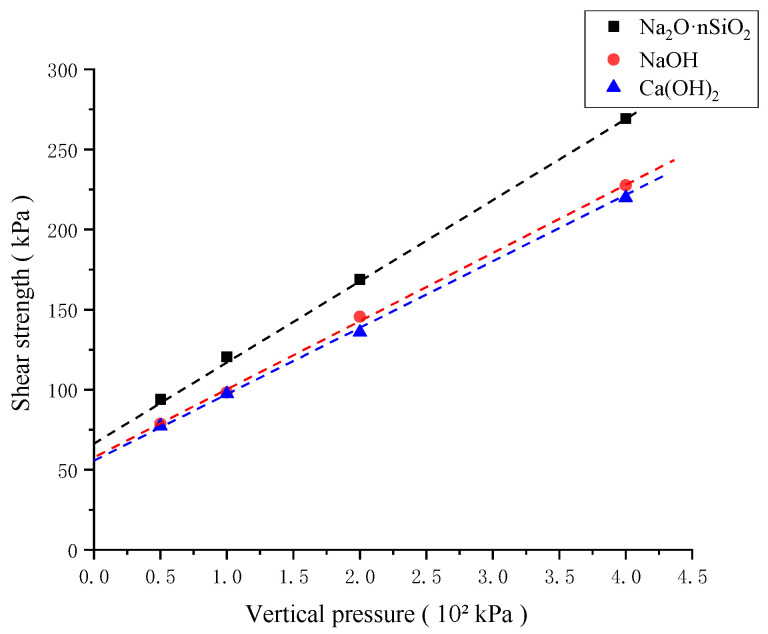
Relation curve of vertical pressure–shear strength.

Figure 7 shows the shear strength values of the three AAS-solidified soils under different vertical pressures, and the cohesion and internal friction angles of the three AAS-solidified soils obtained from Figure 7 are shown in Table 9. It can be seen from the chart that under the same vertical pressure condition, AAS-solidified soil with Na_2_O·nSiO_2_ as the activator has the highest shear strength, followed by AAS-solidified soil with NaOH as the activator and AAS-solidified soil with Ca(OH)_2_ with the worst shear strength.

#### 3.3.4. Compression Modulus

**Figure 8:**
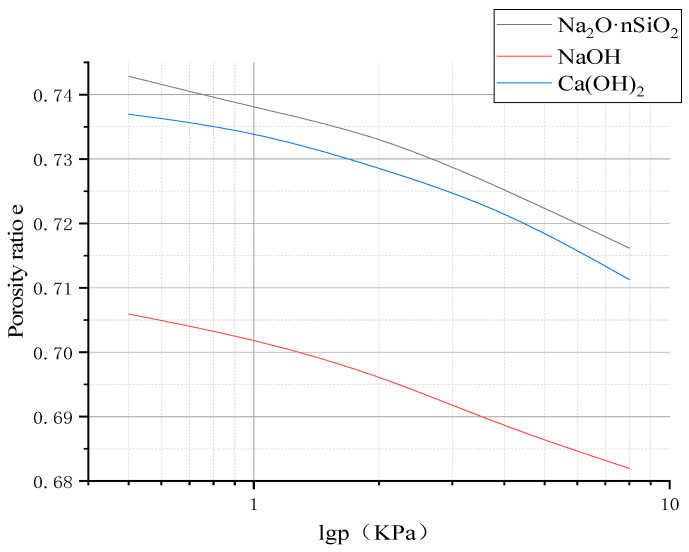
e-lgp curves of three AAS-solidified soils.

Rapid consolidation tests were carried out on three kinds of AAS-solidified soil, and e-lgp curves of AAS-solidified soil under different activators were obtained, as shown in Figure 8.

**Figure 9:**
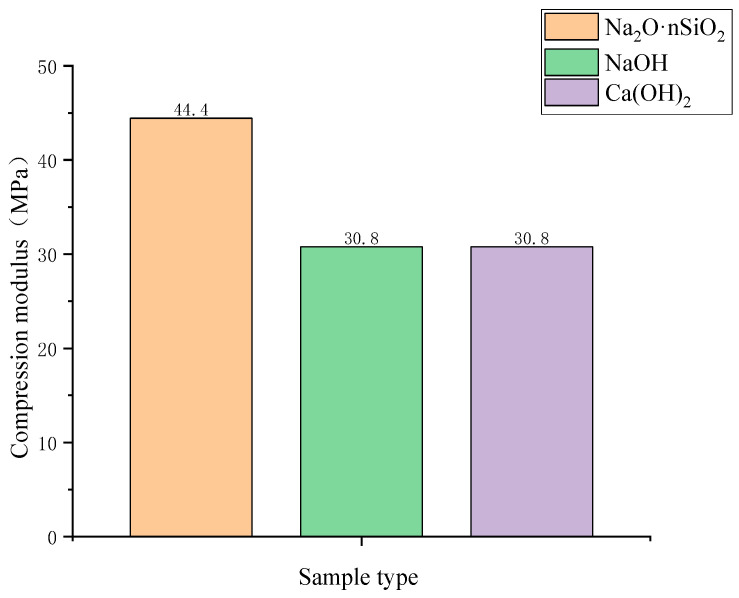
Compression modulus values under different activators.

The compression modulus Es_1-2_, which was obtained when the pressure interval increased from P_i_ = 0.1 MPa to P_i+1_ = 0.2 MPa, was selected to determine the compression characteristics of the solidified soil, and the compression modulus of the three AAS-solidified soils was obtained, as shown in Figure 8. It can be seen from Figure 9 that when the activator is Na_2_O·nSiO_2_, the compression modulus of AAS-solidified soil under the same load is significantly greater than that of AAS-solidified soil under the other two activators; that is, the vertical deformation of AAS-solidified soil under the same load is small.

#### 3.3.5. Evaluation of Macroscopic Mechanical Properties of AAS-Solidified Soil

**Figure 10:**
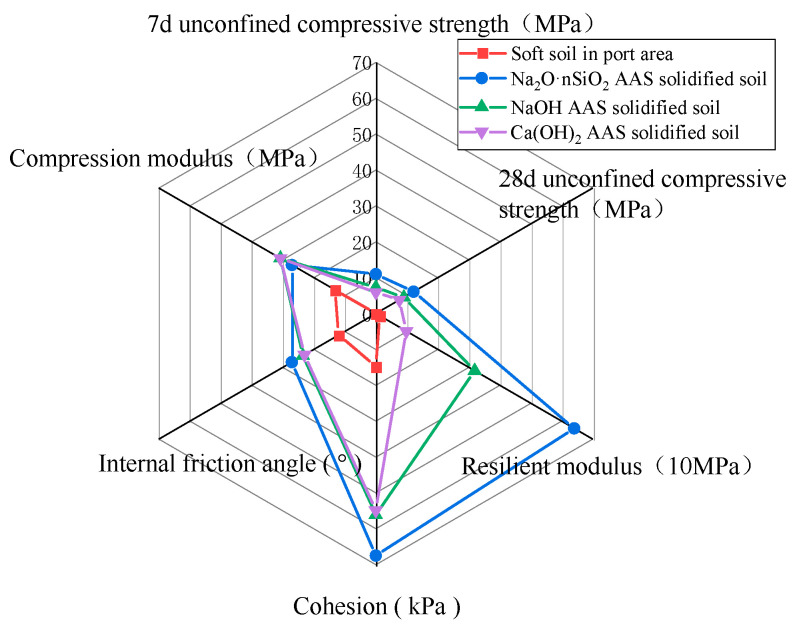
Radar diagram of three AAS-solidified soils.

The macroscopic mechanical properties of AAS-solidified soil and port soft soil were compared separately, as shown in Figure 10. It can be seen from the figure that the three AAS-solidified soils have obvious advantages in improving the unconfined compressive strength of plain soil, especially the unconfined compressive strength of Na_2_O·nSiO_2_ AAS-solidified soil is higher. Regarding resilience modulus, Na_2_O·nSiO_2_ AAS-solidified soil increased by more than 50 times, NaOH AAS-solidified soil increased by more than 20 times, and Ca(OH)_2_ AAS-solidified soil increased by nearly 8 times compared with port soft soil. The compression modulus of the soil cured by AAS is also improved effectively. Compared with the soft soil in the port area, the Na_2_O·nSiO_2_ AAS-solidified soil is increased 4 times, and the NaOH and the Ca(OH)_2_ AAS-solidified soil are both increased 3 times. The internal friction angle and cohesion value of the three AAS-solidified soils are also higher than that of the port soft soils. In general, compared with three kinds of AAS-solidified soil, Na_2_O·nSiO_2_ AAS-solidified soil has the best compressive properties, shear properties, and deformation resistance.

### 3.4. Microscopic Analysis

#### 3.4.1. Phase Composition Analysis

**Figure 11:**
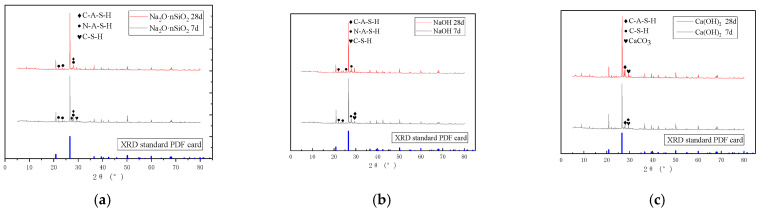
XRD patterns of AAS-solidified soil with different activators. (**a**) Na_2_O·nSiO_2_ (**b**) NaOH (**c**) Ca(OH)_2_.

Three different AAS-solidified soils with curing ages of 7 d and 28 d were analyzed through XRD tests. Figure 11 shows the XRD patterns of the three AAS-solidified soils at different curing ages. The blue line at the bottom of the figure is the PDF card of quartz (SiO_2_), and the diffraction peak of SiO_2_ is removed. The diffraction patterns of the three AAS-solidified soils mainly showed three kinds of sharp crystal peaks: the characteristic peaks of Albite, Anorthite, and Calcium silicate. At the same time, a broad dispersion peak appeared at 25~35°.

It can be seen from Figure 11a that the steamed bun peak (amorphous peak) appears in the Na_2_O·nSiO_2_ AAS-solidified soil at 25~35° at the age of 7 d. C(N)-(A)-S-H is an amorphous gel, and its diffraction peak cannot be seen directly on the XRD pattern, but the mineral powder as a curing agent contains a large amount of CaO, SiO_2_, Al_2_O_3_, and the activator contains Na_2_O. The reaction between the two forms of calcium aluminosilicate hydrate (C-A-S-H) gel, sodium aluminosilicate hydrate (N-A-S-H) gel, and calcium silicate hydrate (C-S-H) gel. Therefore, in the three figures, the steamed bun peak at 25~35° is most likely C(N)-(A)-S-H. Whether it is the gel product mentioned above still needs to be determined by EDS, FTIR, and other analytical means. In addition, in Figure 11a, the characteristic peak of C-S-H did not appear obviously when the curing age was 28 d, which was due to the large amount of Al in the AAS system at the later stage of hydration, which replaced Si in C-S-H, Na in Na_2_O·nSiO_2_ replaced Ca, and generated more C(N)-A-S-H gels. In Figure 11b, when the activator is NaOH, the curing reaction and hydration products are similar to Na_2_O·nSiO_2_. In Figure 11c, when the activator is Ca(OH)_2_, there is only the distinctive peak of C(A)-S-H in the spectrum, and the characteristic peak of CaCO_3_ appears, so the hydration products when the activator is Ca(OH)_2_ are mainly C-(A)-S-H gel and CaCO_3_.

#### 3.4.2. SEM-EDS Analysis

(1) Micromorphology

**Figure 12:**
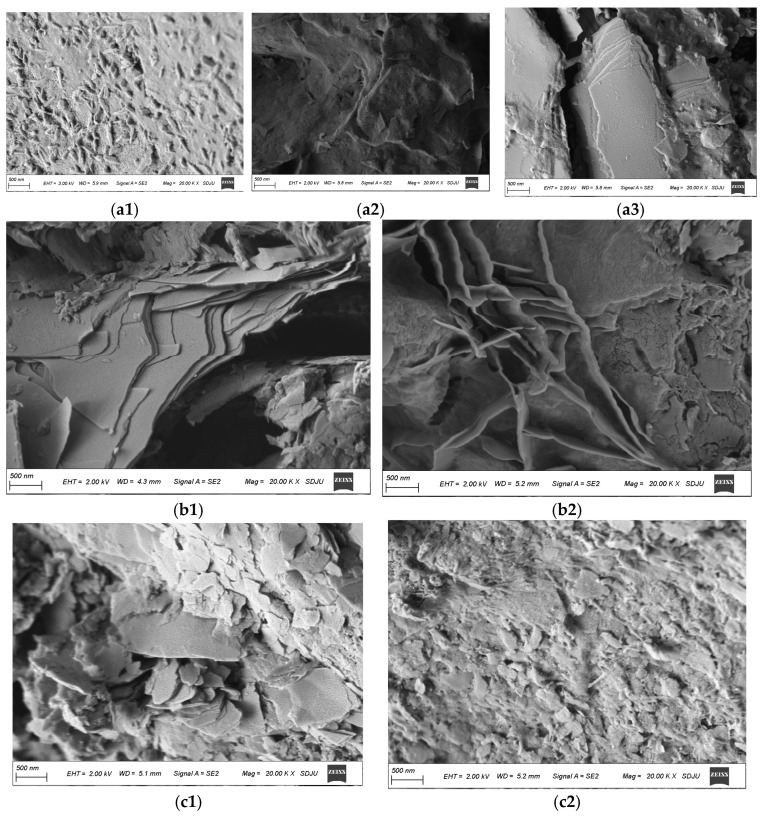
Micromorphologies of different AAS-solidified soils ((**a1**–**a3**): Na_2_O·nSiO_2_ as the activator; (**b1**,**b2**): NaOH as the activator; (**c1**,**c2**): Ca(OH)_2_ as the activator).

Figure 12 shows the SEM images of the products of three AAS-solidified soils enlarged by 20,000 times. As can be seen from the figure, in the soil cured by AAS, the gels generated by the reaction are amorphous or fibrous. These gels form a dense network structure covering the surface of the soil particles and filling the pores, changing the pores from large connected pores to small isolated ones, reducing the porosity of the soil sample. In addition, the gel products are closely bonded to the soil particles, and the interface bonding state is good. Figure 12a1–a3 shows the product morphology of Na_2_O·nSiO_2_-solidified soil. It can be seen that there are flocculent structures wrapped in soil particles and some hydration products, such as flake, rod, or columnar crystals. The product morphology of NaOH AAS-solidified soil is shown in Figure 12b1,b2. The gel products with flake structures are filled in the soil particle gap, and some network structures are interwoven in the cracks. The hydration products of Ca(OH)_2_ are shown in Figure 12c1,c2. The products are polygonal sheets and contain some rod-like hydration products. However, from the image, the gel products do not completely wrap the edges of clay minerals, and there is a phenomenon of falling off.

(2) Element content

**Figure 13:**
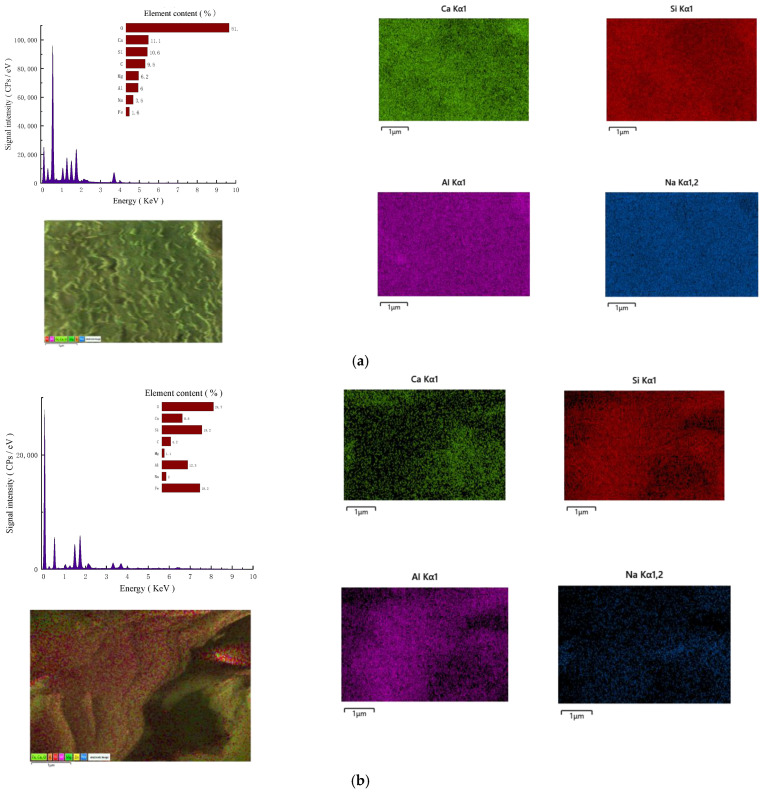

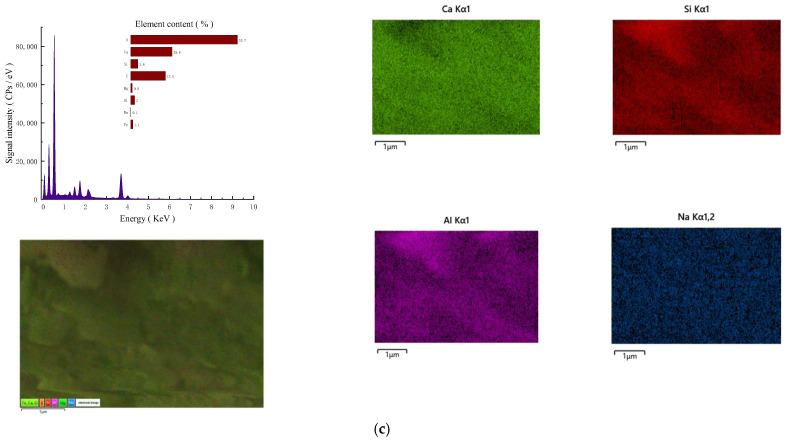
EDS spectra of three AAS-solidified soils. (**a**) Elemental composition and energy spectrum when Na_2_O·nSiO_2_ is the activator. (**b**) Elemental composition and energy spectrum when NaOH is the activator. (**c**) Elemental composition and energy spectrum when Ca(OH)_2_ is the activator.

Figure 13 shows the EDS hydration products of AAS-solidified soil corresponding to Figure 12. The elements are mainly O, Ca, Si, C, Mg, Al, Na, Fe, etc. In Figure 13, the two elements with the highest content are O and Ca, consistent with the XRF results of slag powder, confirming that the hydration products that enhance the strength of soil samples are AAS.

As shown in Figure 13, it can be seen that the regions of Na (blue), Ca (green), Si (red), and Al (pink) elements are highly overlapped, and the content of these elements is relatively high. The flaky and rod-shaped gel products co-located by the three elements are C(N)-A-S-H gel. Filling the gel in the gap can wrap the soil particles, greatly reducing the porosity and cementing the soil particles together well. Compared with Figure 13b,c, the Ca/Si value and Na/Si value of Figure 13a are higher, which means that more C(N)-A-S-H gels are generated. Therefore, the Na_2_O·nSiO_2_ AAS-solidified soil shows better macroscopic mechanical properties than NaOH and Ca(OH)_2_ AAS-solidified soil.

#### 3.4.3. TG-DTG Analysis

**Figure 14:**
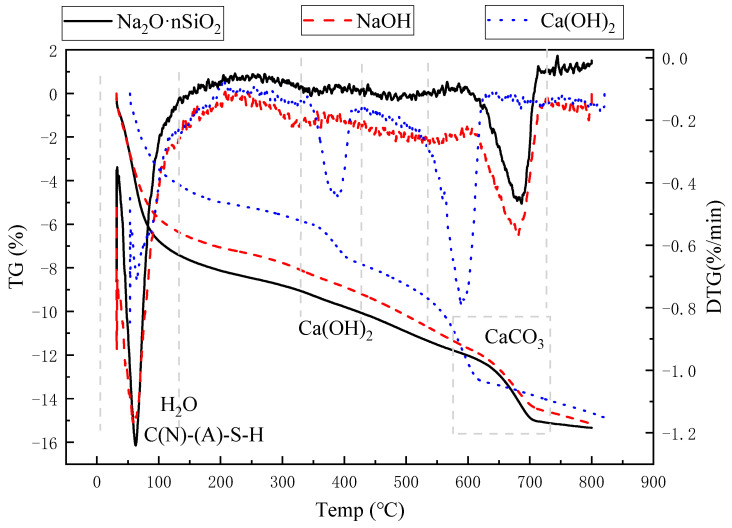
TG-DTG curve of 7 d AAS-solidified soil.

Figure 14 shows the TG-DTG curves (thermogravimetric differential curves) of three AAS-solidified soils at a curing age of 7 d, recording the weight loss of AAS-solidified soils with a temperature of 30~800 °C. In the figure, the most obvious mass loss occurs between 30 °C and 150 °C, which is due to the loss of free water in the solidified soil and the adsorbed water in C-A-S-H gel, N-A-S-H gel, and C-S-H gel. The weight loss of AAS-solidified soil with Na_2_O·nSiO_2_ as the activator is significantly greater than that of the solidified soil with the other two activators. This means that when Na_2_O·nSiO_2_ is the activator, more C(N)-(A)-S-H gels are generated. The second noticeable mass loss occurs between 600 and 800 °C, which is the decomposition of carbonate. There is also an insignificant weight loss peak, occurring between 350 and 450 °C, which is the decomposition of Ca(OH)_2_, and it is more noticeable when the activator is Ca(OH)_2_, indicating that there is Ca(OH)_2_ that does not participate in the reaction.

#### 3.4.4. FTIR Analysis

**Figure 15:**
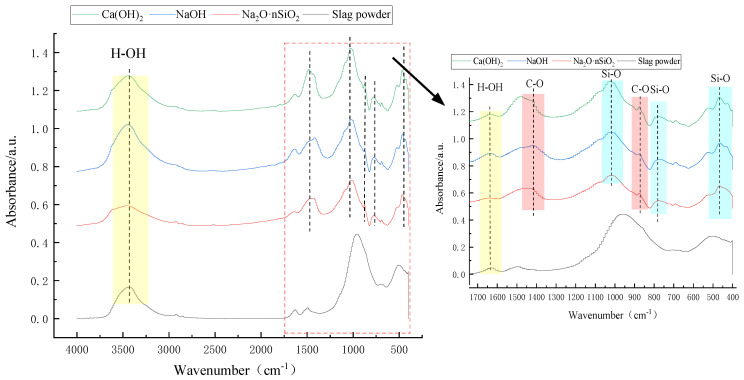
Infrared spectra of slag and 28 d AAS-solidified soil.

Figure 15 shows the infrared spectra of slag powder and AAS-solidified soil. The O–H bond expansion vibration peak of the hydroxyl group in slag powder and AAS-solidified soil is at 3440 cm^−1^, and the bending vibration peak of the O–H bond is at 1650 cm^−1^, indicating that water exists in both slag powder and solidified soil, which is consistent with the TG test results. An amount of 400–1300 cm^−1^ is the fingerprint region of the infrared spectrum, and the characteristics of the absorption peak are relatively strong. Upon magnification observation, the absorption peak of slag powder appears at 500 cm^−1^ and 956 cm^−1^. Additionally, 500 cm^−1^ is the bending vibration peak of the Al–O bond, and 956 cm^−1^ is the stretching vibration of Si–O. The absorption peaks of AAS-solidified soil appeared near 465 cm^−1^, 775 cm^−1^, 875 cm^−1^, 1020 cm^−1^ and 1420 cm^−1^. The absorption peaks at 465 cm^−1^, 775 cm^−1^, 1020 cm^−1^ are caused by the vibration of Al–O and Si–O, which proves that the hydration products of alkali-activated cementitious materials are C-A-S-H and N-A-S-H because they all contain tetrahedral [SiO_4_]^4−^ and [AlO_4_]^5−^. An amount of 1420 cm^−1^ and 875 cm^−1^, corresponding to the asymmetric stretching vibration and bending vibration of the C–O bond, respectively, reflect the formation of silicate.

Compared with sodium silicate AAS-solidified soil, the high-pH environment of NaOH AAS-solidified soil can accelerate the absorption of CO_2_ and generate more carbonates (the peak intensity of C–O bonds increases), which significantly aggravates the degree of carbonization. The increase in O–H peak intensity indicates that the structure of solidified soil is loose and the porosity is higher, which leads to the lower strength and higher permeability of sodium hydroxide AAS-solidified soil. Therefore, an appropriate modulus of Na_2_O·nSiO_2_ can balance the alkalinity supply and silicon source, which is better than that of single-doped sodium hydroxide. In comparing the three kinds of AAS-solidified soil, Na_2_O·nSiO_2_ AAS-solidified soil has a high gel polymerization degree, a small carbonization risk, and the highest mechanical strength potential; NaOH AAS-solidified soil has a high early strength and weak long-term performance; and the micro- and macro-mechanical angles of calcium hydroxide are general.

#### 3.4.5. AAS Reaction Mechanism

Slag is dominated by an amorphous glass structure. The hydration reaction of alkali-activated slag is a multi-stage dynamic evolution process, and its core lies in the depolymerization–reconstruction mechanism of aluminosilicate glass. The specific reaction mechanism can be divided into three stages:

(1) Vitreous deconstruction stage

The OH^−^ in the alkaline environment attacks the bridge oxygen bond, destroys the Si–O-Si and Al–O-Al bonds, and leads to network depolymerization. [SiO_4_] and [AlO_4_] tetrahedrons in the slag undergo a hydrolysis reaction to release the active aluminosilicate monomers [SiO_4_]^4−^ and [AlO_4_]^5−^.

(2) Monomer polycondensation stage

The dissolved monomers [SiO_4_]^4−^ and [AlO_4_]^5−^ coordinate with Ca^2+^ to form a short-chain C-S-H gel.

(3) Gel evolution stage

With the development of the reaction process, the system undergoes the following structural evolution: the aluminum–oxygen tetrahedron enters the silicon–oxygen chain (T-O-T, T = Si/Al) through isomorphism substitution, and the silicon–aluminum monomer forms a cross-linked structure through a polycondensation reaction to form a three-dimensional network structure C-A-S-H gel. Na_2_O·nSiO_2_ and Na^+^ in NaOH replace some calcium sites through ion exchange to form a C(N)-A-S-H composite gel, which further optimizes the structure and mechanical properties of the soil particles.

## 4. Conclusions

(1) Orthogonal tests were carried out on three factors: the type of activator, the amount of activator, and the amount of slag powder in AAS-solidified soil. It was found that the main factor affecting the unconfined compressive strength of AAS-solidified soil was the type of activator, the secondary factor was the amount of activator, and the tertiary factor was the amount of slag powder. The optimal factors were as follows: Na_2_O·nSiO_2_ as the activator type, 3% for the activator amount, and 20% for the slag powder amount.
(2) The unconfined compressive strength, resilience modulus, shear strength, and compression modulus of the three AAS-solidified soils were tested. It was concluded that the effects of the initiators can be ordered as follows: Na_2_O·nSiO_2_ > NaOH > Ca(OH)_2_. Here, the unconfined compressive strength of Na_2_O·nSiO_2_ AAS-solidified soil can reach 11 MPa after 7 days, and the early strength forms quickly. Regarding macroscopic mechanical properties, AAS-solidified soil can effectively improve the mechanical properties of plain soil, and AAS-solidified soil as a foundation has good engineering characteristics.
(3) Through XRD, FTIR, and TG-DTG tests on AAS-solidified soil, it was concluded that the hydration products of AAS are mainly C-A-S-H gel, N-A-S-H gel, and C-S-H gel. The gel cementation of soil particles can enhance the strength and stability of soft soil foundations.

## Figures and Tables

**Table 1:** Appearances of the raw materials.

Soil sample		Slag powder	
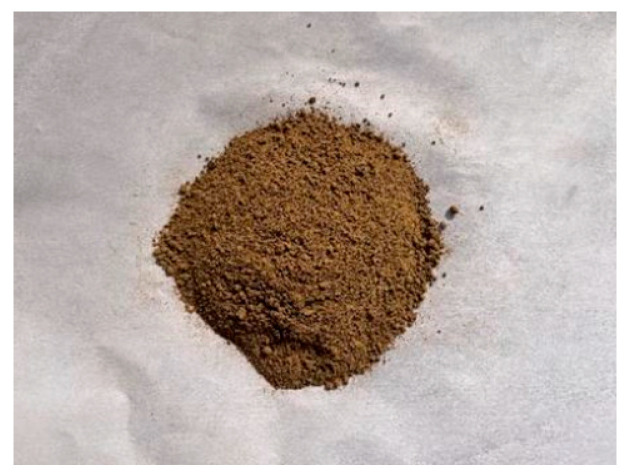		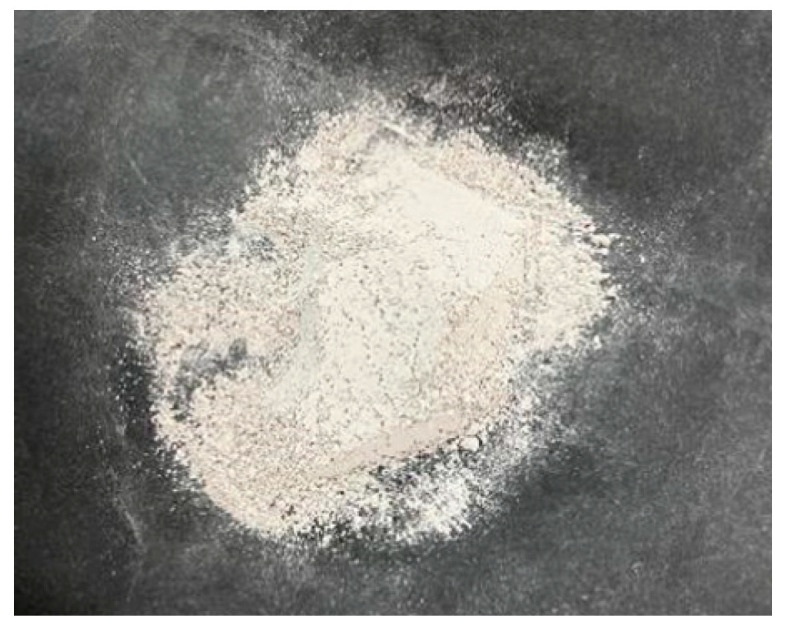	
Na_2_O·nSiO_2_	NaOH		Ca(OH)_2_
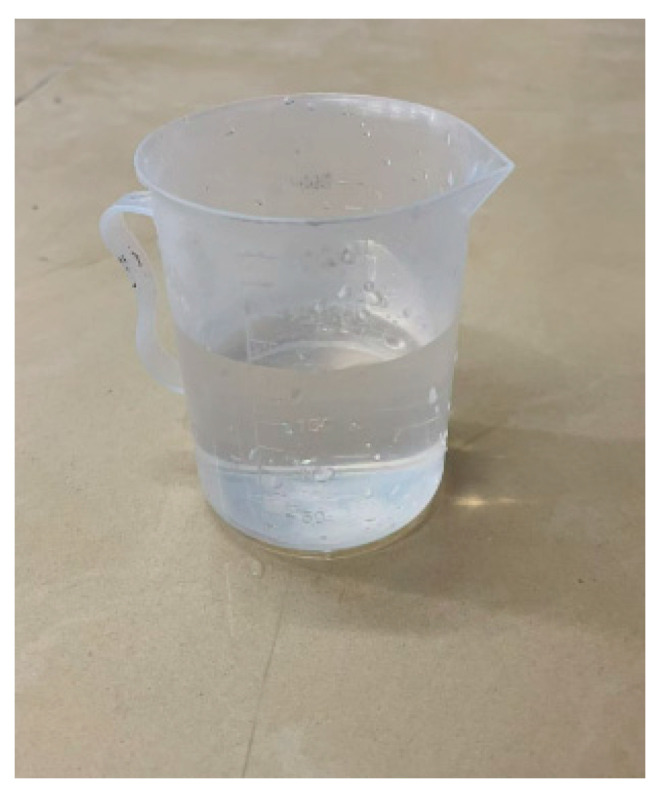	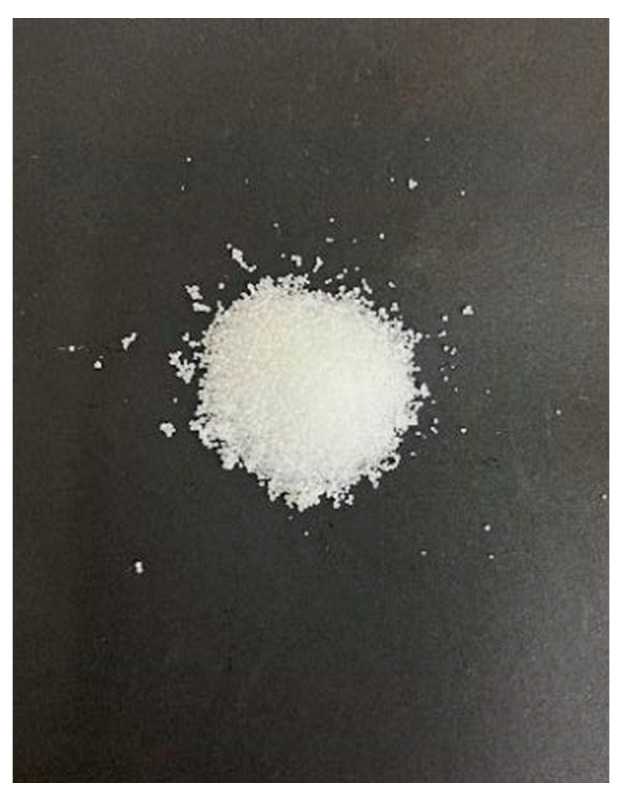		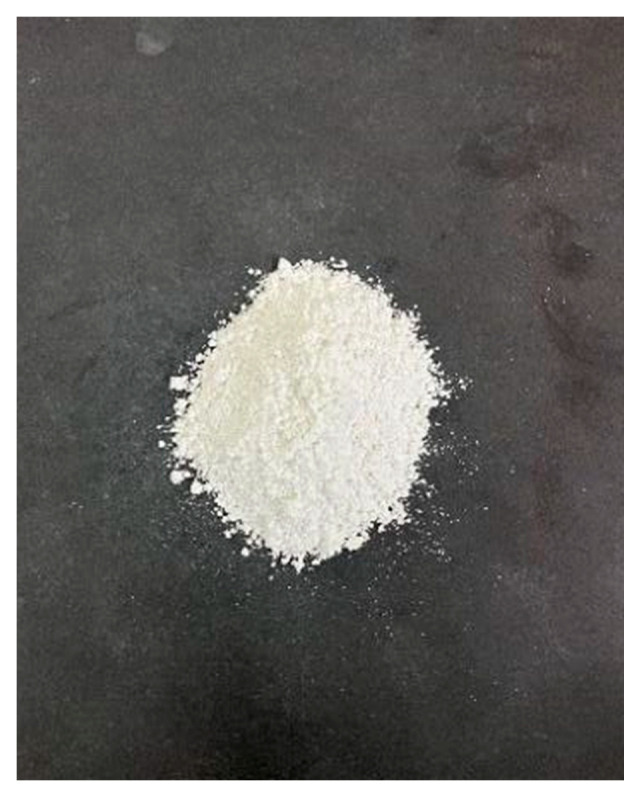

**Table 2:** Physical and mechanical properties of port soft soil.

Natural Moisture Content/%	Maximum Dry Density/g/cm^3^	Best Moisture Content/%	Liquid Limit/%	Plastic Limit/%	Plastic Index
27.05	1.77	11.7	30.61	18.47	12.14

**Table 3:** Oxide content of slag powder.

Oxide	CaO	SiO_2_	Al_2_O_3_	MgO	SO_3_	TiO_2_	Fe_2_O_3_	MnO	K_2_O	Na_2_O
Content/%	37.17	26.66	17.29	12.18	3.42	1.51	0.58	0.37	0.31	0.29

**Table 4:** Basic properties of Na_2_O·nSiO_2_.

Modulus (nSiO_2_/nNa_2_O)	Solid Content/%	SiO_2_/%	Na_2_O/%	Baume Degree/°Bé	PH	Bulk Density/t/m^3^
3.3	35.5	26.98	8.53	38.5	10–13	1.35

**Table 5:** Na_2_O·nSiO_2_ modulus test design.

Na_2_O·nSiO_2_ Modulus	Activator Content/%	Slag Powder Content/%
0.3/0.5/0.7/0.9/1.1	4	15

**Table 6:** Orthogonal test design table of AAS-solidified soil.

No.	Activator Type A	Activator Content B/%	Slag Powder Content C/%
1	Na_2_O·nSiO_2_	5%	20%
2	Na_2_O·nSiO_2_	4%	10%
3	Na_2_O·nSiO_2_	3%	15%
4	NaOH	5%	10%
5	NaOH	4%	15%
6	NaOH	3%	20%
7	Ca(OH)_2_	5%	15%
8	Ca(OH)_2_	4%	20%
9	Ca(OH)_2_	3%	10%

**Table 7:** Orthogonal test results of AAS-solidified soil.

Orthogonal Test Number	7 d Unconfined Compressive Strength	28 d Unconfined Compressive Strength
1	9.159	10.958
2	5.851	9.399
3	11.004	12.188
4	6.304	7.682
5	6.764	6.643
6	9.535	10.781
7	3.753	6.025
8	5.304	7.368
9	5.913	7.272

**Table 8:** Range analysis table.

Range	7 d Unconfined Compressive Strength			28 d Unconfined Compressive Strength		
	Activator Type A	Activator Content B/%	Slag Powder Content C/%	Activator Type A	Activator Content B/%	Slag Powder Content C/%
K1	26.01	26.45	18.07	32.55	30.24	24.35
K2	22.60	17.92	21.52	25.11	23.41	24.86
K3	14.97	19.22	24.00	20.67	24.67	29.11
k1	8.67	8.82	6.02	10.85	10.08	8.12
k2	7.53	5.97	7.17	8.37	7.80	8.29
k3	4.99	6.41	8.00	6.89	8.22	9.70
Superior level	A1	B1	C3	A1	B1	C3
R	3.68	2.84	1.98	3.96	2.28	1.58

**Table 9:** Shear strength index of AAS-solidified soil with different activators.

Type of Solidified Soil	Cohesion C (KPa)	Internal Friction Angle φ (°)
Na_2_O·nSiO_2_ AAS-solidified soil	67.45	27.1
NaOH AAS-solidified soil	56.13	23.5
Ca(OH)_2_ AAS-solidified soil	54.88	23.0

## Data Availability

The original contributions presented in this study are included in the article. Further inquiries can be directed to the corresponding authors.

## Nomenclature

XRF	X-Ray Fluorescence Spectrometer
XRD	X-Ray Diffraction
SEM	Scanning Electron Microscopy
EDS	Energy Dispersive Spectrometer
FTIR	Fourier Transform Infrared Spectrometer
TG	Thermal Gravimetric Analyzer
d	Day
